# RIC in COVID-19—a Clinical Trial to Investigate Whether Remote Ischemic Conditioning (RIC) Can Prevent Deterioration to Critical Care in Patients with COVID-19

**DOI:** 10.1007/s10557-021-07221-y

**Published:** 2021-06-25

**Authors:** Sean M. Davidson, Kishal Lukhna, Diana A. Gorog, Alan D. Salama, Alejandro Rosell Castillo, Sara Giesz, Pelin Golforoush, Siavash Beikoghli Kalkhoran, Sandrine Lecour, Aqeela Imamdin, Helison R. P. do Carmo, Ticiane Gonçalez Bovi, Mauricio W. Perroud, Mpiko Ntsekhe, Andrei C. Sposito, Derek M. Yellon

**Affiliations:** 1 The Hatter Cardiovascular Institute, University College London, 67 Chenies Mews, London, WC1E 6HX UK; 2 Division of Cardiology, Groote Schuur Hospital and Faculty of Health Sciences, University of Cape Town, Cape Town, South Africa; 3 Postgraduate Medicine, University of Hertfordshire, UK & East and North Hertfordshire NHS Trust, Stevenage, Hertfordshire UK; 4 Department of Renal Medicine, Royal Free Hospital, London, UK; 5 Atherosclerosis and Vascular Biology Laboratory, State University of Campinas, Campinas, Brazil; 6 The Hatter Institute for Cardiovascular Research, University of Cape Town, Cape Town, South Africa

**Keywords:** COVID-19, Remote ischemic conditioning, Clinical trial, Cytokines, Immunosuppression, Endothelial cells

## Abstract

**Purpose:**

Coronavirus disease 19 (COVID-19) has, to date, been diagnosed in over 130 million persons worldwide and is caused by severe acute respiratory syndrome coronavirus 2 (SARS-CoV-2). Several variants of concern have emerged including those in the United Kingdom, South Africa, and Brazil. SARS-CoV-2 can cause a dysregulated inflammatory response known as a cytokine storm, which can progress rapidly to acute respiratory distress syndrome (ARDS), multi-organ failure, and death. Suppressing these cytokine elevations may be key to improving outcomes. Remote ischemic conditioning (RIC) is a simple, non-invasive procedure whereby a blood pressure cuff is inflated and deflated on the upper arm for several cycles. “RIC in COVID-19” is a pilot, multi-center, randomized clinical trial, designed to ascertain whether RIC suppresses inflammatory cytokine production.

**Methods:**

A minimum of 55 adult patients with diagnosed COVID-19, but not of critical status, will be enrolled from centers in the United Kingdom, Brazil, and South Africa. RIC will be administered daily for up to 15 days. The primary outcome is the level of inflammatory cytokines that are involved in the cytokine storm that can occur following SARS-CoV-2 infection. The secondary endpoint is the time between admission and until intensive care admission or death. The in vitro cytotoxicity of patient blood will also be assessed using primary human cardiac endothelial cells.

**Conclusions:**

The results of this pilot study will provide initial evidence on the ability of RIC to suppress the production of inflammatory cytokines in the setting of COVID-19.

**Trial Registration:**

NCT04699227, registered January 7th, 2021.

## Introduction

### COVID-19

Coronavirus disease 19 (COVID-19) emerged in late 2019 and has since been diagnosed in well-over 100 million persons worldwide and resulted in over 3 million deaths. It is caused by severe acute respiratory syndrome coronavirus 2 (SARS-CoV-2). The case fatality varies fairly widely from < 0.1 to > 20% according to country but averages ~ 4% worldwide [1]. The disparity may be partly explained by variants of SARS-CoV-2 that have arisen in certain countries, including the (20I/501Y.V1) variant in the United Kingdom, which increases viral transmissibility [2], and the South African (20H/501Y.V2) and Brazilian (20 J/501Y.V3) variants harboring the E484K mutation, which appears to confer heightened resistance to antibody neutralization [3].

### Inflammatory Response and ARDS

SARS-CoV-2 infection can cause a dysregulated inflammatory response known as a cytokine storm, which can progress rapidly to acute respiratory distress syndrome (ARDS), multi-organ failure, and death [4]. Suppressing these cytokine elevations may therefore be key to improving outcomes.

SARS-CoV-2 can cause marked cardiac injury. An elevated plasma level of troponin (cTn) is seen in 20–30% of patients hospitalized with COVID-19 and is associated with greater disease severity and worse outcome [5]. However, despite some evidence that it is possible for SARS-CoV-2 to infect cardiomyocytes [6], fulminant myocarditis is relatively rare in COVID-19. On the other hand, the presence of ARDS is associated with greater cardiac injury [5], which suggests that excess levels of cytokines may be causally involved in the cardiac injury. It is not well understood whether high levels of inflammatory cytokines are *markers*, *mediators*, *or both*, of COVID-19 severity and if these pathways account for cardiovascular impairment [7]. This knowledge may be relevant to the prevention of cardiovascular pathophysiology in SARS-CoV-2 infection.

In addition to cardiac effects, the vasculature is also affected in COVID-19, both directly by the SARS-CoV-2 virus and indirectly as a result of a systemic inflammatory cytokine storm [8]. A hyperinflammatory and pro-coagulatory state is characteristic of COVID-19, which suggests that there is a critical role for the endothelium, both as an effector contributing to inflammation and thrombosis and as a target organ, whose dysfunction may contribute to poor outcome [8]. Therefore, measurements of endothelial dysfunction could be useful in the risk stratification of COVID-19 patients [8].

Developing new methods to reduce the heightened inflammatory response is essential to halting progression of COVID-19 in patients and reducing the severity of damage [4]. Tocilizumab and other IL-6-neutralizing agents can be useful in interrupting the cytokine storm. However, their benefit in hospitalized COVID-19 patients is often modest [9]. Alternative approaches are therefore required that can suppress the cytokine response more broadly.

### Remote Ischemic Conditioning

Remote ischemic conditioning (RIC) is a simple, non-invasive procedure in which a blood pressure cuff is applied to the upper arm for repeated cycles of inflation and deflation (typically 3–5 cycles of 5 min each) [10, 11]. This process activates pro-survival mechanisms in the body to protect vital organs and improve the immune system [11–14]. Therefore, we believe it represents a promising strategy to protect organs against reduced blood flow and heightened immune response, as seen in patients with COVID-19 infections [13]. Several studies suggest that RIC can suppress cytokine induction in the setting of cardiac surgery or sepsis [12, 13, 15, 16].

The above observations that cytokine storm contributes to COVID-19 morbidity and mortality and that RIC can suppress the inflammatory response led us to propose that RIC could provide benefit in patients with COVID-19 [4, 13]. The rationale of this study is thus that RIC will supress cytokine induction in patients with COVID-19, leading to less cytokine-induced cardiac injury.

### Study Hypothesis

We propose to test the hypothesis that RIC, via repeated cycles of inflation and deflation of a peripheral arm blood pressure cuff, administered daily, reduces inflammation in COVID-19 patients.

### Study Objectives

The “RIC in COVID-19” study will be a proof-of-concept study to investigate the effect of RIC in COVID-19 patients. The study objective is to determine whether RIC reduces the level of inflammatory cytokines in non-critically ill patients admitted to hospital with COVID-19. Primary and secondary outcome measures are detailed in the “Study Endpoints” section.

## Methods

### Study Design

“RIC in COVID-19” is a pilot, multi-center, randomized study designed to ascertain whether RIC decreases the severity of inflammatory markers associated with a “cytokine storm” score.

The study has been reviewed and approved with a favorable opinion from the United Kingdom National Research Ethics Service (NRES) (South Central – Berkshire Research Ethics Committee reference: 20/SC/0192). The study protocol is registered on the public trials database clinicaltrials.gov NCT04699227. This study has also received approval by the Ethics Committee of the State University of Campinas (CAAE: 33,709,320.4.0000.5404) and by the Human Research Ethics Committee, University of Cape Town, South Africa (HREC 407/2020).

### Study Population

A minimum of 55 non-critical adult patients with a confirmed diagnosis of COVID-19 will be consecutively enrolled into the study. The patients will be identified as those admitted to either The Royal Free (London UK), The Lister Hospital (Stevenage, UK), Hospital Estadual Sumaré (Sumaré, Brazil), or Groote Schuur Hospital (Cape Town, South Africa). After enrolment, patients will be randomized (n = 30 per group) in a 1:1 fashion to receive either RIC or sham-control intervention.

Patients will be included according to the following criteria:

- Adult patients (≥ 18 to 80 years of age) with a diagnosis of COVID-19 infection, confirmed by positive PCR reaction.
- Clinical features of respiratory distress, i.e., with respiratory rate > 30 bpm or use of accessory muscles; or
- Peripheral oxygen saturation SaO2 < 95% or requiring oxygen supplementation, or
- Clinically unwell with reduced mobility, raised temperature/heart rate, and/or deranged biochemical laboratory results.

Patients will be excluded on the basis of any of the following exclusion criteria:

- Contraindication for the use of a brachial cuff on either arm;
- Intercurrent disease with an expected life expectancy of less than 24 h;
- Cardiac arrest;
- Pregnant or breastfeeding women;
- Bleeding disorder or platelet count below 50;
- Currently enrolled in another research study;
- End-stage renal disease requiring renal replacement therapy;
- Chronic liver disease and/or ALT and AST ≥ 5 times the normal upper reference limit;
- Significant immunodeficiency states: AIDS/HIV not on antiretroviral agents, solid organ transplants, bone marrow transplants, chronic use of immunomodulating therapy such as anti-TNF-α or corticosteroids with prednisone-equivalent dose ≥ 20 mg/day prior to COVID diagnosis;
- Any active underlying malignancy;
- Baseline stage C chronic heart failure or symptomatic chronic obstructive pulmonary disease.
- Critical illness requiring invasive ventilation, including patients with:
  - Hemodynamic instability or
  - Signs of severe hypoxemic respiratory failure despite non-invasive ventilation as defined by the following:
    - Respiratory rate > 40 breaths per minute, and
    - Pulse oximetry (SpO2) less than 90%, or
    - Arterial blood sample with PaO2 < 8.0 kPa, or
    - Heart rate > 120 beats per minute

### Trial Intervention

The RIC protocol will consist of 3–4 cycles of cuff inflations to at least 20 mmHg above systolic blood pressure for 5 min with deflation to 0 mmHg for a further 5 min, which will be automatically administered by the pre-programmed CellAegis (Canada), or Segall (Denmark) autoRIC pneumatic device.

Patients randomized to the sham-control group will have 3–4 cycles of automated low-pressure cuff inflation to 20 mmHg for 5 min and deflation to 0 mmHg for a further 5 min, by a visually identical pneumatic device as used in the RIC protocol. Trial intervention will be performed daily up to 15 days or until either patient discharge or deterioration requiring critical care. The trial intervention will not delay or affect the patient’s clinical management in accordance with local center policies.

### Randomization and Blinding

After enrolment, patients will be randomized 1:1 to receive either RIC or sham-control intervention. The research team who will perform the trial intervention will be unblinded as they will need to be informed of which protocol to administer to the patient. All subsequent analysis will be performed blinded to treatment.

### Study Endpoints

The primary outcome is the level of inflammatory cytokines that are involved in the cytokine storm that can occur following SARS-CoV-2 infection. Venous blood will be collected following RIC administration (on the day of admission and every other day subsequently, when possible) and saved for the subsequent measurement of inflammatory markers such as C-reactive protein (CRP), TNF-α, IL-1β, IL-6, and D-dimer, in addition to cardiac biomarkers troponin T and NTpro-BNP.

Secondary endpoints include the following: [i] time to clinical deterioration (defined as time from randomization to mortality or worsening of the World Health Organization (WHO) Clinical Progression Scale — assessed by the increase of 2 points in this scale), [ii] serum IL-6 ≥ 80 pg/mL as a biomarker of severe clinical outcomes in COVID-19 infection, and [iii] cytokine storm score measured by area under the curve (AUC) serum TNF-α, IL-1β, IL-6, and CRP. The effect of plasma from COVID-19 patients on cardiac endothelial cells will also be evaluated as a secondary endpoint by measuring the survival of primary cardiac endothelial cells incubated with patient plasma [17].

### Sample Size Calculations

Since there are no data available on the effect of RIC on the inflammatory response in COVID-19 patients and the objective of this pilot study is not to provide a formal assessment of efficacy, the sample size was established empirically in 55 individuals in a conservative expectation of a small standardized effect size (0.2).

### Statistical Analysis

Summary statistics will be provided by treatment group for demographic and baseline characteristics, which will be compared among treatment groups for the ITT population. Between-group differences in demographic and baseline characteristic will be tested using a chi-square test (for categorical variables) or a 1-way analysis of variance (ANOVA) model with treatment as a factor (for continuous variables). The significance of these tests will be used as an initial assessment for satisfactoriness of randomization. For continuous variables, changes from baseline to subsequent days of follow up will be considered outcomes. These outcomes will be compared according to the treatment groups adjusted to the baseline values using covariance analysis models (ANCOVA) or by rank analysis of covariance (RANCOVA) according to their distribution. The normality distribution will be investigated using normality tests such as histograms, kurtosis, asymmetry, Kolmogorov–Smirnov, and Shapiro–Wilk in order to select the appropriate tests to compare the data.

### Data Management, Governance, and Funding

The trial is funded by the Thomson Family Charitable Trust and the Hatter Cardiovascular Institute and sponsored by University College London and by the Fundação de Apoio a Pesquisa do Estado de São Paulo (FAPESP). Data will be collected on a case report form (CRF) and managed using REDCap electronic data capture tools. REDCap [18] is a secure, web-based application designed to support validated and audited data capture for research studies. An independent data monitoring committee will be convened to monitor the progress and safety of the study.

## Discussion

While the immune response is clearly essential in the host response to infection, an extreme immune response leading to a cytokine storm can cause collateral damage to the host, that is greater than the immediate benefit of the immune response [19]. Monocytes and macrophages are particularly enriched in the lungs of COVID-19 patients and appear to be important in the pathogenicity of the disease. SARS-CoV-2 infection alters monocyte metabolism leading to inhibition of the T-cell response and reduction of epithelial cell survival [20].

There is no single cytokine that can provide a specific indication of a cytokine storm, and the precise response can vary depending on the infective agent. The first-line host defense to viral infections such as SARS-CoV-2 is provided by the innate immune system. This produces type I interferon, proinflammatory cytokines (e.g., IL-6 and TNF-α), and chemokines. Stimulation of the NLRP3 inflammasome further activates caspase-1, which cleaves pro-IL-1β and pro-IL-18 into active proinflammatory cytokines. These can activate NF-κB to augment IL-6 and TNF-α expression. Thus, interferon-γ, interleukin-1, interleukin-6, TNF-α, and interleukin-18 are key cytokines that are often found to be elevated in cytokine storm situations and are believed to have central immunopathologic roles [19]. Both IL-1 and IL-6 are critical in regulating CRP levels, which has been found to be prognostic with regard poor outcome in patients with COVID-19 [21]. In terms of the response to SARS-CoV-2 infection, an in-depth transcriptomic analysis revealed an imbalanced inflammatory response defined by low levels of type I and III interferons with elevated chemokines and high expression of IL-6 [22]. This would suggest that controlling inflammation is more important than targeting the IFN response in treatments for COVID-19.

The primary aim of this study is to investigate the effect of RIC on cytokine production and inflammatory responses in COVID-19. Several animal studies have shown that RIC suppresses cytokine induction via downregulation of NF-κB, a central transcription factor mediating proinflammatory gene induction in both innate and adaptive immune cells, and myeloperoxidase (MPO) pathways (reviewed in [13]). Two of the largest clinical trials to measure cytokines in patients who had been administered RIC (with n = 65, n = 90 participants) demonstrated cytokine attenuation in the treatment group undergoing RIC prior to off-pump CABG and colorectal surgery, respectively. In the latter study, levels of IL-1β and TNF-α were significantly reduced [13].

A cytokine storm-like response with excessive cytokine release can also occur in other setting such as sepsis [19]. Acute and chronic (repeated daily) RIC has been shown to provide benefit following LPS-induced sepsis in animal models [12, 15]. In these studies, RIC reduced the levels the proinflammatory cytokines TNF-α, IL-1β, and IL-6, as well as suppressing the sepsis-induced increase in plasma cardiac troponin I [12, 15]. Furthermore, mortality was significantly reduced following chronic RIC administration as opposed to acute RIC administration [12, 15]. RIC has also been seen to improve survival in sheep with sepsis [16]. RIC has not been extensively evaluated in patients with sepsis, but a small clinical trial saw an improvement in microcirculatory flow in septic patients administered RIC [23].

Measurements of endothelial dysfunction could be useful in the risk stratification of COVID-19 patients [8]. Plasma from critically ill COVID-19 patients causes rapid and direct cytotoxicity in an in vitro assay using human primary pulmonary endothelial cells [17]. Endotheliopathy is a central part of the pathological response to severe COVID-19 and may contribute to cardiac injury [24]. In the “RIC in COVID-19” study, the effect of plasma from COVID-19 patients will also be evaluated on primary cardiac endothelial cells incubated with patient plasma with the aim of determining whether the endothelium may be directly affected in COVID-19.

In conclusion, this pilot study will allow us to ascertain whether RIC has a part to play in reducing the overall markers of inflammation and secondary outcomes.

## Data Availability

Data will be made available on reasonable request.

## Abbreviations

ARDS: Acute respiratory distress syndrome
COVID-19: Coronavirus 2019
CRF: Case report form
cTn: Troponin
RIC: Remote ischemic conditioning
SARS-CoV-2: Severe acute respiratory syndrome coronavirus 2
